# Functional diversity and habitat preferences of native grassland plants and ground‐dwelling invertebrates in private gardens along an urbanization gradient

**DOI:** 10.1002/ece3.8343

**Published:** 2021-11-18

**Authors:** Brigitte Braschler, José D. Gilgado, Hans‐Peter Rusterholz, Sascha Buchholz, Valerie Zwahlen, Bruno Baur

**Affiliations:** ^1^ Section of Conservation Biology Department of Environmental Sciences University of Basel Basel Switzerland; ^2^ Department of Ecology Technische Universität Berlin Berlin Germany; ^3^ Berlin‐Brandenburg Institute of Advanced Biodiversity Research (BBIB) Berlin Germany

**Keywords:** body size, domestic gardens, dry‐adapted species, functional dispersion, functional evenness, urban ecology

## Abstract

Urbanization is occurring around the globe, changing environmental conditions and influencing biodiversity and ecosystem functions. Urban domestic gardens represent a small‐grained mosaic of diverse habitats for numerous species. The challenging conditions in urban gardens support species possessing certain traits, and exclude other species. Functional diversity is therefore often altered in urban gardens. By using a multi‐taxa approach focused on native grassland plants and ground‐dwelling invertebrates with overall low mobility (snails, slugs, spiders, millipedes, woodlice, ants, rove beetles), we examined the effects of urbanization (distance to city center, percentage of sealed area) and garden characteristics on functional dispersion, functional evenness, habitat preferences and body size. We conducted a field survey in 35 domestic gardens along a rural–urban gradient in Basel, Switzerland. The various groups showed different responses to urbanization. Functional dispersion of native grassland plants decreased with increasing distance to the city center, while functional dispersion of ants decreased with increasing percentage of sealed area. Functional evenness of ants increased with increasing distance to the city center and that of rove beetles decreased with increasing percentage of sealed area. Contrary to our expectation, in rove beetles, the proportion of generalists decreased with increasing percentage of sealed area in the surroundings, and the proportion of species preferring dry conditions increased with increasing distance to the city center. Body size of species increased with distance to city center for slugs, spiders, millipedes, ants, and rove beetles. Local garden characteristics had few effects on functional diversity and habitat preferences of the groups examined. Our study supports the importance of using multi‐taxa approaches when examining effects of environmental change on biodiversity. Considering only a single group may result in misleading findings for overall biodiversity. The ground‐dwelling invertebrates investigated may be affected in different ways from the more often‐studied flying pollinators or birds.

## Graphical abstract

Private gardens are assumed to play an essential role in ecosystem functioning in cities. We examined whether functional diversity, body size and habitat preferences of native plants and several groups of invertebrates in gardens change along an urbanization gradient.

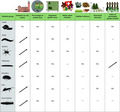

## 1 INTRODUCTION

Urbanization is currently one of the major drivers of global change (Güneralp et al., 2020) with multifarious consequences for biodiversity and ecosystems (McDonald et al., 2020). Among others, negative impacts derive from habitat loss and fragmentation due to the increase of impervious surfaces and barrier effects of the urban structure (Beninde et al., 2015; Fenoglio et al., 2020), an increase in temperature due to the urban heat island effect (Hamblin et al., 2018; Piano et al., 2017; Youngsteadt et al., 2017), and direct anthropogenic disturbance (Delgado de la flor et al., 2019). Despite these negative effects, cities may also offer unexploited opportunities for biodiversity (Samways et al., 2020; Soanes & Lentini, 2019). For example, urban habitats can serve as secondary habitats or refuges, respectively, for rare and endangered species, whose primordial habitats are degraded by the intensification of agriculture or even destroyed (Hall et al., 2016; Ives et al., 2016).

Urban gardens constitute an important part of urban green space and despite their small size, they cover large parts of urban area (Gaston et al., 2013). Home or community gardens are distributed throughout the city and provide valuable stepping‐stones for numerous species and thus are an essential part of the urban green infrastructure (Cameron et al., 2012). At a local scale, gardens provide a heterogeneous small‐grained mosaic of diverse habitats formed through different user management practices and individual owner preferences (Braschler et al., 2021; Lin & Egerer, 2020; Smith et al., 2005). Gardens may harbor a considerable variety of vascular plants, which in turn increases biodiversity of herbivores and decomposers (Adams et al., 2020). Therefore, invertebrate biodiversity of urban gardens could be remarkable as has been shown for cities in the United Kingdom (Smith, Chapman, & Eggleton, 2006; Smith, Gaston, et al., 2006), the United States (Egerer et al., 2017; Philpott et al., 2014), and Switzerland (Braschler et al., 2020).

While studies on biodiversity aspects in cities have increased for certain taxa (e.g., spiders, ground beetles or pollinators including wild bees; Martins et al., 2017; Piano, Bona, & Isaia, 2020; Piano, Souffreau, et al., 2020; Threlfall et al., 2015), other, often less conspicuous taxa have rarely been the focus of urban biodiversity research (e.g., millipedes, rove beetles, for an exception see Smith, Chapman, & Eggleton, 2006; Smith, Gaston, et al., 2006). Multi‐taxon studies considering urban gardens are even rarer (but see, e.g., Braschler et al., 2020; Egerer et al., 2017; Tresch, Frey, Le Bayon, Mäder, et al., 2019; Tresch, Frey, Le Bayon, Zanetta, et al., 2019). Furthermore, studies on the effects of the urban matrix on functional diversity and the distribution of biological traits across several taxa are scarce (but see Melliger et al., 2017). This may impede our understanding of biodiversity changes along urbanization gradients as functional approaches incorporating species traits provide a mechanistic understanding of how species communities are shaped by the environment (Scheiner et al., 2017). In this context, analyzing relationships between species’ biological traits and environmental factors can help to identify which species can thrive in certain urban habitats (Wong et al., 2019). For example, urban habitats can act as environmental filters for species because some biological traits may be beneficial for inhabiting cities, while others may lead to the segregation of species (Buchholz & Egerer, 2020).

Building on this concept, we aimed to investigate how functional diversity is changing along urbanization gradients and which combination of biological traits is favored in highly urbanized areas. We examined these questions using a multi‐taxon approach focusing on native grassland plants and several groups of ground‐dwelling invertebrates (snails, slugs, spiders, millipedes, woodlice, ants, and rove beetles). As measures of functional diversity we considered functional dispersion (FDis) and functional evenness (FEve). FDis is a measure of functional richness, which considers the species’ relative abundances by estimating their dispersion in a multi‐dimensional trait space (Laliberté & Legendre, 2010). FEve describes the evenness of abundance distribution in a functional trait space.

In our study, we tested the following four hypotheses:

1. Due to increased homogenization, which is often assumed for urban environments (Knop, 2016), we expected that functional diversity expressed as functional dispersion (Laliberté & Legendre, 2010) will decrease with increasing urbanization. Similarly, we expected that functional evenness, which measures the regularity of the distribution of species in functional space (Mason et al., 2005), will also be reduced, as only a few species sharing similar traits can cope with urban conditions. However, these species may then become very abundant.
2. Based on the assumption that in cities habitat generalist species are favored (Concepción et al., 2015, 2016), we expected an increase of habitat generalists in urban habitats.
3. The urban heat island causes higher temperatures in cities when compared with rural surroundings. Many thermophilic species also show preference for dry conditions (xerothermophilic species) (Horváth et al., 2012; Seifert, 2007). Following Menke et al. (2011) and Piano et al. (2017), who reported an enhanced proportion of thermophilic ant and carabid species respectively in urban sites, and Horváth et al. (2012), who reported higher numbers of xerophilic spider species in urban forest sites than in suburban or rural forest sites, we hypothesized that the abundance of dry adapted species will increase with increasing urbanization and that this will be consistent across all examined groups. This might be the case in spite of frequent irrigation in most private gardens along the urbanization gradient. We expected more xerothermophilic species in gardens within highly urbanized surroundings, although irrigation may partly dampen the urban heat island effect in some microhabitats of the gardens.
4. Within a taxonomic group, increased temperature may lead to a decline in body size (Gardner et al., 2011). Several studies provided evidence that this can result in small‐sized species being more numerous in urban assemblages, as shown in bumblebees (Eggenberger et al., 2019), wild bees (Hamblin et al., 2018), spiders, carabids and weevils (Merckx et al., 2018) and in habitat specialists among carabids (Magura et al., 2020). Hence, we expected that for some of the examined groups of ground‐dwelling invertebrates the proportion of small‐sized species would increase with degree of urbanization.

## 2 MATERIAL AND METHODS

### 2.1 Garden selection

The study was conducted in the city of Basel, its suburbs and nearby villages in North‐western Switzerland (47˚34'N, 7˚36'E). Total annual precipitation averages 842 mm and annual mean temperature is 10.5°C in the city (records from 1981 to 2010, www.meteoswiss.admin.ch). We selected 35 gardens from a pool of 65 candidates offered in response to public calls. The chosen gardens reflect a rural–urban gradient, encompassing wide ranges of garden sizes and management types. Further criteria for the garden choice were acceptance of the intended sampling methods by the garden owners and guaranteed daytime access to the gardens. All gardens had a grassland area of at least 4 m^2^, allowing us to set up traps and hay baits, but they differed in the composition of other habitat types (see below).

### 2.2 Plant and ground‐dwelling invertebrate surveys

We recorded all native plant species (including woody species) occurring in the grassland by slowly walking in a zigzag line over the grassland area of a garden (hereafter native plant species richness in grassland). Plants were recorded when touching this line. Thus, sampling effort was proportional to the size of the grassland area in a garden. For total native plant species richness used as explanatory variable (see below), we complemented native plant species richness in grassland by recording the native plant species in the other habitat types by slowly walking along transect lines. These lines ran along the long axis of garden features (e.g., flower beds, vegetable plots, and hedges). Furthermore, we considered plants at intervals of 2 m along the transect line to measure the height of the vegetation, which we used to calculate structural diversity of the vegetation as a covariate for models on invertebrate functional diversity. Sampling effort for native plant species richness was thus proportional to the area with vegetation. Plant diversity was assessed in all gardens between July 24 and August 20, 2018.

We surveyed seven groups of ground‐dwelling invertebrates (for details of sampling see Braschler et al., 2020). The groups cover a wide range of feeding strategies and included phylogenetically distant taxa: two groups of Gastropoda (snails and slugs), Araneae (spiders), Diplopoda (millipedes), Isopoda (woodlice), Formicidae (ants), and Staphylinidae (rove beetles) excluding the subfamily Pselaphinae. Some years ago, the Pselaphinae was considered a distinct family (Newton & Thayer, 1995), therefore our expert did not determine this group. Members of this group have a very different morphology from other rove beetles. Furthermore, very little information on habitat preferences was available for this subfamily. We used pitfall traps (plastic beakers, 5.8 cm diameter, containing saltwater as a preservative, that were buried flush with the ground) and hay bait traps (moist hay in coarse plastic netting placed in direct contact with the ground; Tuf et al., 2015) to sample all groups. Hay bait traps complement pitfall traps by providing a spot of humid conditions and thus attracting less‐mobile, frequently in the leaf‐litter or upper soil layers living invertebrates, as well as acting as a food resource attracting many detritivores and their predators (Tuf et al., 2015). We placed five pitfall traps and five hay baits in the grassland of each garden. Pitfall traps and hay baits were operated three times for 7 days each from early to late summer 2018. We employed additional techniques for four groups (snails and slugs (active search and sieving of soil samples), millipedes and ants (active search); see Braschler et al. (2020) for details). Invertebrate surveys were conducted between May 31 and October 18, 2018.

In the grassland area of the gardens, we recorded a total of 157 native plant species (Braschler et al., 2020). For spiders and woodlice, we only considered adult individuals and for ants, only workers. In the remaining invertebrate groups, we included all individuals that could be determined to the species level (99.4% of all individuals sampled). References for identification keys used and nomenclature followed can be found in Braschler et al. (2020). In all, we considered 1744 snail individuals (34 species), 1671 slug individuals (6 species), 1079 adult spider individuals (52 species), 6864 millipede individuals (21 species), 2582 adult woodlice individuals (9 species), and 1265 individuals of rove beetles (85 species). Furthermore, we found 28 species of ants (abundance data for this group was not considered because of the aggregated nature of ant colonies).

### 2.3 Traits and habitat preferences

As measures of functional diversity, we considered functional dispersion (FDis) and functional evenness (FEve). FDis is a measure of functional richness, which considers the species’ relative abundances by estimating their dispersion in a multi‐dimensional trait space (Laliberté & Legendre, 2010). FDis has no upper limit, and high values correspond to large numbers of functionally different species. FEve describes the evenness of abundance distribution in a functional trait space. High FEve‐values indicate a balanced niche occupancy, which occurs in species communities with balanced trait frequencies (Mason et al., 2005). To calculate FDis and FEve, we used morphological and life‐history traits and microhabitat preference of the species recorded in the various gardens. The set of traits varied among groups (Table 1).

**TABLE 1:** Plant and invertebrate traits used in the analyses. Measures of body length were used both for calculating FDis and FEve and as a dependent variable in analyses of the effects of urbanization on body size. Not all traits or habitat preferences were available for all taxonomic groups^a^

Trait	Type	Specification	References
(a) Traits used to calculate FDis and FEve^a^			
Plants			
Life form	Categorical	Macrophanerophyte; nanophanerophyte; chamaephyte; hemicryptophyte; geophyte; therophyte	1
Reproduction type	Categorical	Sexual; mixed; self‐fertilization	1
Ecological strategy	Categorical	C; CR; CS; CSR; S; SR; R (categories after Grime, 1979)	1
Pollination syndrome	Categorical	Insects; wind	1
Seed dispersal type	Categorical	Zoochory; anemochory; hemerochory; autochory; hydrochory	2
Seed mass	Continuous	Mean of seed mass (mg)	1
Snails			
Shell size	Continuous	The longer of shell height or shell breadth (mm)	3
Age at sexual maturity	Ordinal	<1 year; 1 year; >1 year	4–6
Longevity	Ordinal	<1 year; 1–2 years; >2 years	5, 6
Shell shape (snails)	Categorical	Depressed; globose/conical; oblong	3
Spiders			
Body size	Continuous	Mean male body length (mm)	7
Hunting mode	Categorical	Ambush; ground hunter; orb web; sensing web; sheet web; space web; specialist; other	8
Shading width	Continuous	Measure for the niche width	9
Humidity width	Continuous	Measure for the niche width	9
Millipedes			
Body length	Continuous	Mean of male and female body length of first adult stage (mm)	10–15
Breadth	Continuous	Maximum body breath at mid length of individual (mm)	10–15
Eye morphology	Continuous	Number of ocelli	10–13, 16
Feeding guild	Categorical	Detritivore; facultative scavenger; algivore	10–12, 17
Ants			
Body size	Continuous	Maximum total length of workers, including major workers in species where these forage (mm)	18–21
Main food	Categorical	Carbohydrates; animal matter; carbohydrates & animal matter; grains	18–20
Main nest stratum	Categorical	Wood & litter; soil & crevices; both	18–20
Colony founding mode	Categorical	Independent; social parasite	18–20
Number of queens	Categorical	Monogynous; polygynous (species where only some nests have more than one queen are considered polygynous)	18–20
Rove beetles			
Body size	Continuous	Mean of mean body length of males and females (mm)	10, 22–30
Microhabitat preference	Categorical	No specific microhabitat preference; saprophilous; coprophilous; thermophilous but no further preference; hygrophilous but no further preference; phytodetricolous	31
(b) Measures for habitat preferences			
Native grassland plants			
Habitat preference	Binominal	Habitat specialist (grassland); habitat generalist (occurring in two or more habitat types, e.g., open land, forest or agricultural land)	32
Preference for dry conditions	Binominal	Preference for wet or moist habitats (indicator value above 3); preference for dry habitats (indicator value up to 3)	33
Snails			
Habitat preference	Binominal	Habitat specialist (openland or forest); habitat generalist	6
Spiders			
Habitat preference	Binominal	Habitat specialist (openland or forest); habitat generalist	34
Humidity preference	Binominal	Wet or moist preferring; dry preferring	34
Millipedes			
Habitat preference	Binominal	Habitat specialist (openland or forest); habitat generalist	11–13, 35–37
Woodlice			
Habitat preference	Binominal	Habitat specialist (openland or forest); habitat generalist	38–42
Humidity preference	Binominal	Wet or moist preferring; dry tolerant	38–42
Ants			
Habitat preference	Binominal	Habitat specialist (openland or forest); habitat generalist	18
Humidity preference	Binominal	Preference for wet or moist habitats (indicator value above 3.5); preference for dry habitats (indicator value up to 3.5)	18
Rove beetles			
Habitat preference	Binominal	Habitat specialist (openland or forest); habitat generalist	31, 43–45
Humidity preference	Binominal	Wet or moist preferring; dry tolerant	43–45

Note

Sources: 1 Klotz et al. (2002); 2 Müller‐Schneider (1986); 3 Kerney et al. (1983); 4 Bengtsson and Baur (1993); 5 Baur (1994); 6 Falkner et al. (2001); 7 Nentwig et al. (2021); 8 Cardoso et al. (2011); 9 Entling et al. (2007); 10 own data, J. D. Gilgado; 11 Anderson (1996); 12 Blower (1985); 13 Gregory et al. (2015); 14 Haacker (1968); 15 Read et al. (2002); 16 Peitsalmi and Pajunen (1992); 17 Hopkin and Read (1992); 18 Seifert (2007); 19 https://www.antwiki.org; 20 https://ameisenwiki.de; 21 Kutter (1977); 22 Ádám (2010); 23 Lee and Ahn (2017); 24 Salnitska and Solodovnikov (2019); 25 Stan (2007); 26 Webster et al. (2016); 27 http://coleonet.de/coleo/index.htm; 28 https://www.kaefer‐der‐welt.de/index.htm; 29 https://www.ukbeetles.co.uk/index; 30 https://www.kerbtier.de; 31 data B. Feldmann; 32 Delarze et al. (2015); 33 Ellenberg (2001); 34 Hänggi et al. (1995); 35 Kime and Enghoff (2011); 36 Kime and Enghoff (2017); 37 Pedroli‐Christen (1993); 38 Farkas and Vadkerti (2002); 39 Harding and Sutton (1985); 40 Legrand (1948); 41 Schultz (1965); 42 Sutton (1972); 43 Luka (2004); 44 Luka et al. (2010); 45 Luka et al. (2013).

^a^ For woodlice only information on the trait body size (mean adult length in mm) was available. Similarly, for slugs we only considered body size (extended body length in mm). FDis and FEve were therefore not calculated for these groups.

Urbanization alters environmental conditions (Gilbert, 1989; Sukopp & Wittig, 1998) and increases frequency, magnitude and type of disturbance (Niemelä, 2011). This may be to the detriment of some habitat specialists and thus increase the proportion of generalist species. We therefore divided species in those that are habitat generalists and those that are specialized to a particular habitat type (Table 1). Similarly, we divided species into those showing a preference for dry conditions and those showing either a preference for moist or wet conditions or without a preference (Table 1). In the various invertebrate groups, body size is measured in a different way (Table 1).

### 2.4 Local garden characteristics and landscape variables

We assessed 11 local garden characteristics: total garden area, area with vegetation, grassland area, percentage of grassland in a garden, area of shrubs and trees, percentage shrub and tree cover in a garden, habitat richness, length of non‐permeable garden border, percentage length of non‐permeable garden border, index of permeable garden border. In addition, for analyses of invertebrate functional diversity, we used the two characteristics total native plant species richness and structural diversity of the vegetation as explanatory variables. However, due to collinearity, we omitted several of the above variables from the data analyses, retaining only total native plant species richness, habitat richness, structural diversity and index of permeable border (Table 2).

**TABLE 2:** Definitions of local garden characteristics and landscape variables and transformation of data in the analyses. For details of methods, see Braschler et al. (2020)

	Unit	Transformation for analyses^a^	Description
Garden size			
Total garden area	m^2^	Log	Total garden area excluding buildings
Vegetated garden area	m^2^	Log	Area covered by any type of vegetation, including semi‐sealed areas
Garden habitat diversity			
Habitat richness	count	^b^	Summed occurrence of nine defined habitat features
Structural diversity^c^	Shannon index	^b^	Shannon diversity of height of trees and shrubs, and plants in grassland, flower and vegetable beds
Naturalness			
Total native plant species richness^c^	count	^b^	Number of native plant species in the area with vegetation
Isolation of gardens			
Index of permeable garden border	%	Not transformed	Index combining weighted length of permeable and semi‐permeable garden border expressed as percentage of total border length
Landscape variables			
Percentage of sealed area	%	Log	Percentage of sealed area in a radius of 200 m around the garden
Distance to city center	m	Log	Distance from the garden to the town hall of Basel city

^a^ Log‐transformed for GLM analyses.

^b^ Residuals of the relationship variable–total garden area were used for GLM models.

^c^ Used as explanatory variable in the analyses of invertebrate FDis, FEve and body size in invertebrates.

We could not measure management intensity in a precise way for the following reasons. Firstly, some garden owners have changed in the recent past. The history of their gardens’ management (e.g., previous herbicide and pesticide applications) was thus not known. Secondly, within gardens the management is spatially heterogeneous (e.g., intensively managed flowerbeds and lawn with nearby wild‐growing hedges and high‐turf grassland). Our sampling considered all these differently managed habitat types. Thirdly, we received insufficient information on management decisions from the garden owners. For example, most did only provide vague statements on mowing frequency. Similarly, garden owners had quite different views on the meaning of the term “pesticide.” Snail baits were frequently not considered as a poison. Fourthly, turf height, which is sometimes used as a measure related to management intensity, was measured, but was also unreliable as we discovered that several garden owners did mow their lawns in anticipation of our visits. We therefore decided to use the number of native plant species as a proxy for naturalness of the gardens in models analyzing FDis and FEve of invertebrates (see below). We assume that the naturalness of a garden is inversely related to the management intensity of the garden.

As landscape variables we determined for each garden the percentage of sealed area and percentage of green area in the surroundings (both within a radius of 200 m) and the distance to the city center (Table 2). In data analyses, we considered only percentage of sealed area and distance to the city center because percentage of green area was correlated with both.

### 2.5 Statistical analyses

Statistical analyses were performed in R (ver. 3.3.3 and ver. 3.6.1; R Core Team, 2015) and were carried out separately for the different taxonomic groups with the 35 gardens as replicates. We used various traits, depending on the plants or the invertebrate group examined, to calculate FDis and FEve (Table 1) using the FD package in R (Laliberté & Legendre, 2010). For native grassland plants and for ants, we used presence/absence data, and for the remaining groups we used abundance data to calculate FDis and FEve. We applied generalized linear models (GLM) with Gaussian error distribution to examine potential effects of landscape variables, garden size (vegetated garden area) and various local garden characteristics on species richness of different taxonomic groups. The three main variables (distance to city center, percentage sealed area and vegetated garden area) were retained in all models, while a step‐wise procedure was followed with the other four garden characteristics (total native plant richness, structural diversity of the vegetation, habitat type richness and index of permeable border) to obtain the minimal adequate models (Crawley, 2007). Three variables (total native plant richness, structural diversity of the vegetation and habitat type richness) were correlated with total garden area. Therefore, we used residuals of the relationships between the variable and total garden area for the GLM models. Distance to city center and percentage of sealed area were slightly correlated (*r* = −0.46, *p* = .006). However, we retained both in the model because they represent different aspects of urbanization. The same GLM model was used to analyze urbanization‐related effects on body size for invertebrate groups.

To examine whether the proportion of generalist species was affected by urbanization, we used GLM models with the three variables distance to city center, percentage of sealed area within 200 m and vegetated garden area (all log‐transformed). The same model was used to examine the effect of urbanization on the proportion of species showing a preference for dry conditions. We applied a binominal error distribution and logit link function. To detect overdispersion, we compared the residual deviance with the residual degrees of freedom, and if the former were much higher (ratio >> 1), then we used quasi‐binominal error distribution instead (Crawley, 2007).

## 3 RESULTS

### 3.1 Functional dispersion

**FIGURE 1:**
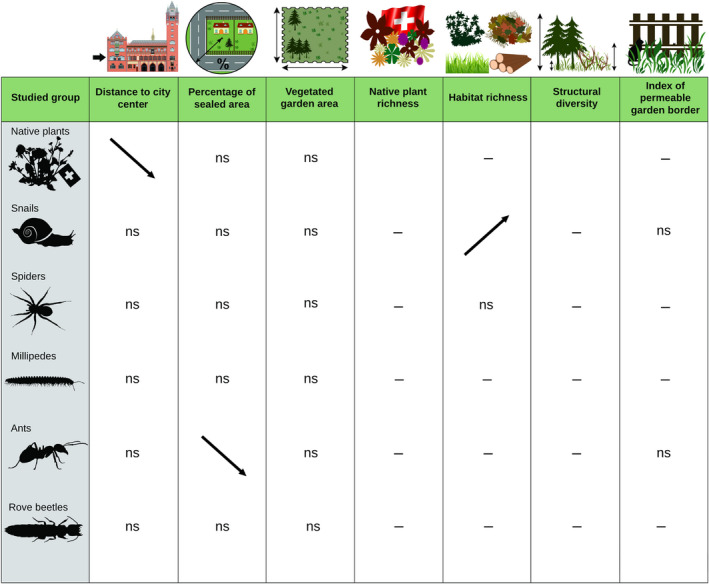
Effects of urbanization, garden size, and local garden characteristics, habitat type richness, structural diversity of the vegetation, and index of permeable border on FDis of native grassland plants and five groups of invertebrates. Arrows indicate the direction of significant effects, “–” indicates that this variable was removed from the model in the stepwise procedure, “ns” refers to variables included in the model but that were not significant. Data were transformed as described in the statistical analyses section and Table 2

In the 35 gardens investigated, we found a negative effect of distance to city center on FDis of native grassland plants (Figure 1; Table 3; Figure S1). Percentage of sealed area negatively influenced FDis of ants (Figure 1; Table 3; Figure S1). No significant effects of distance to city center or percentage of sealed area on FDis were found for the other invertebrate groups. Habitat type richness was the only local garden characteristics to affect FDis, which was increasing in snails with increasing habitat type richness (Figure 1; Table 3; Figure S1).

**TABLE 3:** Summary of GLM analyses examining the effects of two measures of urbanization (distance to city center and percentage of sealed area in the surroundings) and garden size (vegetated garden area), total native plant species richness, habitat type richness, structural diversity of the vegetation, index of permeable border on functional dispersion (FDis) and functional evenness (FEve) of various organism groups

Organism group	Variable	FDis			FEve		
		df	*F*	*p*	df	*F*	*p*
Native grassland plants	Distance to city center^a^	1,33	7.60	.**010**	1,33	2.26	.14
	Percentage sealed area^a^	1,32	.05	.82	1,32	.16	.69
	Vegetated garden area^a^	1,31	.11	.74	1,31	3.64	.066
	Habitat type richness^b^	–	–	–	–	–	–
	Index of permeable border	–	–	–	–	–	–
Snails	Distance to city center^a^	1,33	2.94	.097	1,32	.01	.94
	Percentage sealed area^a^	1,32	.01	.91	1,31	.39	.54
	Vegetated garden area^a^	1,31	.26	.61	1,30	.49	.49
	Total native plant species richness^b^	–	–	–	–	–	–
	Habitat type richness^b^	1,30	5.68	.**024**	1,29	1.05	.31
	Structural diversity of the vegetation^b^	–	–	–	–	–	–
	Index of permeable border	1,29	1.90	.18	–	–	–
Spiders	Distance to city center^a^	1,33	.04	.85	1,33	.43	.52
	Percentage sealed area^a^	1,32	.84	.37	1,32	1.39	.25
	Vegetated garden area^a^	1,31	2.57	.12	1,31	<.01	.99
	Total native plant species richness^b^	–	–	–	1,30	3.76	.062
	Habitat type richness^b^	1,30	3.84	.059	1,29	3.73	.063
	Structural diversity of the vegetation^b^	–	–	–	–	–	–
	Index of permeable border	–	–	–	–	–	–
Millipedes	Distance to city center^a^	1,33	.14	.71	1,30	.89	.35
	Percentage sealed area^a^	1,32	.01	.92	1,29	4.07	.053
	Vegetated garden area^a^	1,31	<.01	.98	1,28	.96	.33
	Total native plant species richness^b^	–	–	–	–	–	–
	Habitat type richness^b^	–	–	–	–	–	–
	Structural diversity of the vegetation^b^	–	–	–	–	–	–
	Index of permeable border	–	–	–	–	–	–
Ants	Distance to city center^a^	1,33	1.16	.29	1,33	16.67	**<.001**
	Percentage sealed area^a^	1,32	4.41	.**044**	1,32	1.58	.22
	Vegetated garden area^a^	1,31	.39	.54	1,31	22.66	**<.001**
	Total native plant species richness^b^	–	–	–	–	–	–
	Habitat type richness^b^	–	–	–	–	–	–
	Structural diversity of the vegetation^b^	–	–	–	–	–	–
	Index of permeable border	1,30	2.75	.11	–	–	–
Rove beetles	Distance to city center^a^	1,33	.29	.59	1,33	.08	.78
	Percentage sealed area^a^	1,32	.09	.76	1,32	6.75	.**014**
	Vegetated garden area^a^	1,31	.32	.58	1,31	.12	.73
	Total native plant species richness^b^	–	–	–	–	–	–
	Habitat type richness^b^	–	–	–	–	–	–
	Structural diversity of the vegetation^b^	–	–	–	–	–	–
	Index of permeable border	–	–	–	1,30	2.68	.11

Note

Significant *p*‐values (<.05) are in bold. FDis and FEve are based on abundance data except for native grassland plants and ants, for which presence/absence data was used. “–” variable was excluded from the model by step‐wise reduction.

^a^ Log‐transformed.

^b^ Due to correlation with total garden size, residuals of the regression of the variable on total garden size were used for analyses.

### 3.2 Functional evenness

**FIGURE 2:**
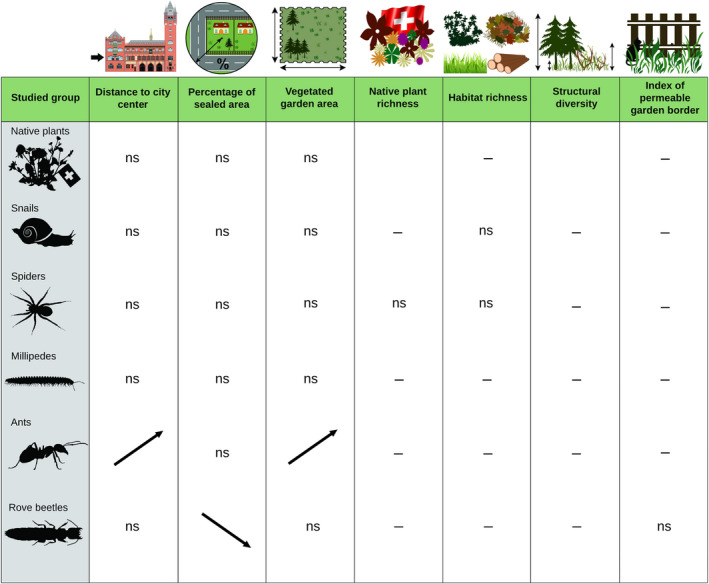
Effects of urbanization, garden size and local garden characteristics, habitat type richness, structural diversity of the vegetation, and index of permeable border, on FEve of native grassland plants and five groups of invertebrates. For detailed explanations, see caption to Figure 1

Distance to city center positively affected FEve of ants (Figure 2; Table 3; Figure S2). In rove beetles, percentage of sealed area within 200 m negatively affected FEve (Figure 2; Table 3; Figure S2). Vegetated garden area positively influenced the FEve of ants (Figure 2; Table 3; Figure S2). Other local garden characteristics did not significantly affect FEve in any group (Figure 2; Table 3; Figure S2).

### 3.3 Habitat preference

The proportion of generalists among rove beetles declined with increasing percentage of sealed area in the surroundings of the gardens in contrast to our hypothesis (Table S1). In the other groups, the proportion of generalists was not affected by either distance to the city center or percentage of sealed area (Table S1).

Also contrary to our expectation, the proportion of rove beetle species preferring dry conditions increased with increasing distance to the city center (Table S1). For the other groups examined neither distance to the city center nor percentage of sealed area influenced the proportion of species preferring dry conditions (Table S1).

### 3.4 Body size

**FIGURE 3:**
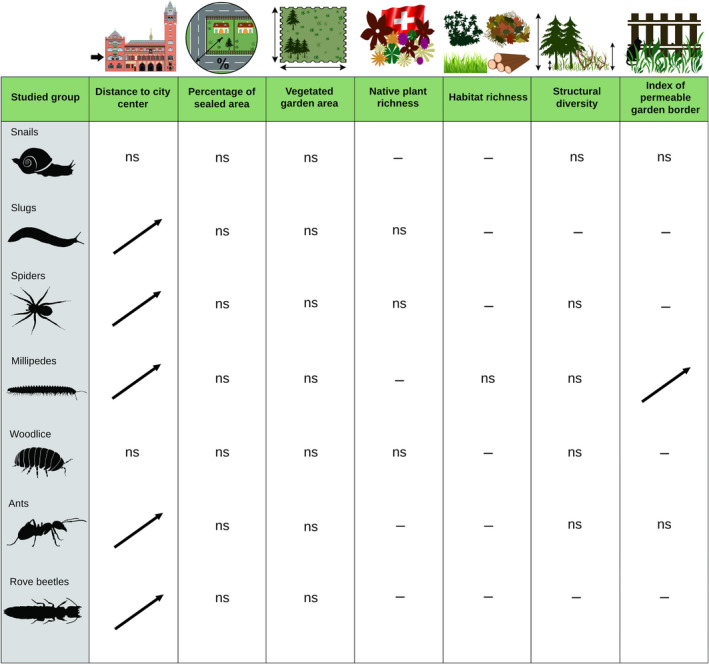
Effects of urbanization, garden size and local garden characteristics, habitat type richness, structural diversity of the vegetation, and index of permeable border, on body size of seven groups of invertebrates. For detailed explanations, see caption to Figure 1

Distance to city center affected body size in all invertebrate groups except snails and woodlice (Figure 3; Table S2; Figure S3). Body size increased with distance to city center for slugs, spiders, millipedes, ants, and rove beetles. In millipedes, body size also increased with index of permeable garden border (Figure 3; Table S2; Figure S3).

## 4 DISCUSSION

Urbanization can affect functional diversity of organisms and the distribution of biological traits (Williams et al., 2009). We used a multi‐taxon approach, including plants and seven groups of ground‐dwelling invertebrates, which revealed that the observed effects of urbanization—either at landscape or at habitat level—can be multifarious and are often ambivalent. In some groups, effects of urbanization on certain aspects of functional diversity may even be positive, while other groups do not show a response to the increasing degree of urbanization at all.

We found no evidence for an overall homogenization in terms of a reduced FDis across the investigated taxa, except for native grassland plants and ants. The responses of FEve were also sporadic and only significant for ants and rove beetles. The first hypothesis is therefore not supported by our findings. The second hypothesis assuming that the proportion of generalists is higher in urban than in rural gardens, could not be confirmed either as the proportion of generalists did not change over the urbanization gradient in all groups except in rove beetles. The latter even showed the opposite trend with a decreased proportion of generalists in urban gardens.

Our third hypothesis assumed that the proportion of xerophilic species is increased in urban gardens due to the urban heat island effect. Our results could, however, not confirm this hypothesis. Contrary to our expectations, only xerophilic rove beetle species responded, but in the opposite direction from our expectation making up a lower proportion of the assemblage in urban gardens. Finally, we found strong evidence confirming our fourth hypothesis that body size decreases with increasing urbanization. In most invertebrate groups examined, the average size of the species decreased with decreasing distance to the city center.

### 4.1 Functional dispersion

The environmental conditions in urban areas can act as filters, which have the potential to reduce plant and invertebrate species richness, change species composition and thus functional diversity (Buchholz et al., 2020; Williams et al., 2009). This does not necessarily lead to a decrease in biodiversity. Urban areas can also harbor high species richness, sometimes higher than in the rural surroundings (Kühn et al., 2004; Wania et al., 2006).

In plants, species composition has been reported to be more homogeneous in urban habitats than rural ones (Zeeman et al., 2017), which may result in a decreased functional diversity. We therefore hypothesized that FDis decreases along the rural–urban gradient. However, we recorded higher FDis in plants in highly urbanized areas than in rural ones. This contrasts the findings from other habitat types. Knapp et al. (2012) and Melliger et al. (2018) demonstrated that FDis in plants decreased with increasing degree of urbanization in grassland and forest. Our unexpected result could be explained by species composition not being affected by distance to the city center. However, the higher FDis of native grassland plant species at short distance to the city center may be due to the observed higher similarity among plant assemblages of the grasslands in the city center than in the rural surroundings (H.‐P. Rusterholz, *unpublished data*). This was mainly caused by gardens in the city center having slightly higher proportions of annual plants in their grasslands than gardens in the rural surroundings (H.‐P. Rusterholz, *unpublished data*). Furthermore, grasslands in gardens are in most cases not naturally grown plant communities (Gilbert, 1989). Seed mixtures and management actions influence plant species composition of grasslands in gardens and thus FDis (Knapp et al., 2012).

Ants were the only group that responded as expected; their FDis decreased with increasing urbanization, expressed as percentage of sealed area in the surroundings. We do not have any explanation for this result, and did not find any literature on ant FDis changes along urbanization gradients. However, species composition of ants is generally considered as a good indicator of altered environmental factors and of land‐use change (Andersen, 2000; Kaspari & Majer, 2000). Therefore, functional groups of ants are frequently considered in assessments of land‐use change (Andersen, 2000). Thus, one may expect that responses to land‐use change accompanying urbanization may be captured by ant FDis, as shown in our study.

For the other invertebrate groups examined we found no effect of urbanization on functional dispersion. Tóth and Hornung (2020) reported decreased FDis with increasing urbanization for millipedes in forests and other types of woodland (e.g., parks) in Budapest, Hungary. The decrease in this other habitat type was mainly due to a reduced number of forest specialist species in highly urbanized areas. This is consistent with the finding of Bogyó et al. (2015) for millipedes in urban forests. Similarly, Nagy et al. (2018) reported that functional diversity (measured as functional richness) in woodlice decreased along the urbanization gradient in forests. However, gardens provide other environmental conditions than urban forests. In contrast to forests, gardens may, independent of their location along the rural–urban gradient, provide relatively similar conditions for ground‐dwelling invertebrates due to similar management (see below).

### 4.2 Functional evenness

Decreasing FEve with increasing urbanization as found for rove beetles may indicate temporally variable habitat conditions due to disturbance or environmental stressors, which cause an unbalanced niche occupancy (Schleuter et al., 2010). This can be a result of the dominance of few species that may have adapted to urban environments, while specialized species may disappear as found for rove beetles by Magura et al. (2013).

Similar to the findings on FDis, FEve of ants changed with urbanization in our study, being lower close to the city center. Additionally, FEve of ants increased with increasing vegetated garden area. Increasing the size of a garden may thus counteract some of the negative effects on FEve, as ant colonies with their durable nests are potentially more severely affected by local garden characteristics than more mobile species. Indeed, ant species richness increased in gardens with larger vegetated area (Braschler et al., 2020).

For the other ground‐dwelling invertebrate groups examined we recorded no effects of urbanization on FEve. This matches the few other studies, which for the most part found no changes in invertebrate FEve along urbanization gradients (e.g., Banaszak‐Cibicka and Dylewski (2021) for bees and Correa et al. (2021) for dung beetles).

### 4.3 Habitat preference

Contrary to our expectations, the proportion of habitat generalists increased in rove beetles in rural gardens, while no change in the proportion of generalists was found in the other groups examined. However, Melliger et al. (2018) reported an increase in the share of habitat generalists with increasing percentage of sealed area in ants and spiders in urban forests in Basel. Similarly, Magura et al. (2013) found that the proportion of forest‐associated rove beetles was significantly lower in urban forest, when compared with suburban and rural forest. Forests, which are less intensively managed than private gardens, may show steeper rural–urban gradients for abiotic environmental factors and/or levels of disturbance. Unlike the more frequently studied forests or grasslands, urban gardens may harbor less species with strong habitat bindings. Urban gardens consist of a mosaic of patches of different habitat types. These patches may be too small to harbor certain habitat specialist species. Furthermore, as a result of regular management activity, variation in some environmental conditions may be dampened in gardens independent of the location of the garden. While we could not directly study the effects of management intensity on functional diversity in the examined gardens, because of challenges including heterogeneous management within gardens and imprecise information provided by owners, our observations gave us no reason to expect garden management to change along the rural–urban gradient. The similar environmental conditions in gardens in both rural and urban settings might be more powerful in filtering species composition than other factors such as the composition of the surrounding matrix, which change along the rural–urban gradient (Braschler et al., 2020). For example, repeated watering excludes pronounced periods of drought. This may also explain why—contrary to our expectations—we did not find an increased proportion of xerophilous species in gardens in highly urbanized areas, despite the existing urban heat island effect. Menke et al. (2011) suggested that urban areas may serve as habitat and corridors for dry‐adapted and heat‐tolerant species, and provided some evidence for this in ants along a rural–urban gradient including different habitat types in Raleight, North Carolina, USA, which is similar in population to the greater Basel area.

In plants, factors other than management including watering may lead to homogenization of species assemblages in gardens along the rural–urban gradient. Importantly, grasslands in gardens typically originate from commercial seed mixtures (Gilbert, 1989). Grassland plant species assemblages in gardens are also strongly affected by mowing regimes and trampling (Bertoncini et al., 2012). Furthermore, grassland plant communities are commonly characterized by a low proportion of generalist species (Ellenberg, 1986). A combination of these factors may explain why we did not find a change in the proportion of generalist plant species in the grasslands of the gardens. Similarly, the percentage of generalist species in the other ground‐dwelling invertebrate groups did not change along the rural–urban gradient.

### 4.4 Body size

We found that urban gardens compared with rural ones harbored more small‐sized species in five out of the seven groups of invertebrates examined. One explanation for this pattern is the well‐known heat island effect, which is also well documented in the city of Basel (Wicki et al., 2018). Increased temperatures may influence the body size distributions both within species and at the species assemblage level with larger species becoming rarer at higher temperatures (Gardner et al., 2011; Verberk et al., 2021). In general, this could be related to the temperature–size rule (Atkinson, 1994), for which an ecological explanation could be a larger requirement for resources (food and oxygen) under warmer conditions preventing animals from growing larger (Verberk et al., 2021). An alternative explanation is that animals consist of smaller cells in warm environments (Verberk et al., 2021). The trend towards smaller body size will continue due to global warming and may lead to significant changes in the diversity and species composition of animals in cities. This in turn will affect body size‐dependent ecosystem services in cities, especially as smaller species may not be as effective predators, decomposers, seed dispersers or pollinators as larger species are.

The decrease in millipede body size with decreasing distance to the city center mirrors the findings from other studies (Bogyó et al., 2015; Tóth & Hornung, 2020). Bogyó et al. (2015) hypothesized that the decreased size in millipedes in urban areas is a result of lower food quality, while Tóth and Hornung (2020) suggested that it may be related to a combination of low food quality, deteriorated soils, reduced soil moisture, and increased soil contamination. For example, reduced soil moisture is likely to affect smaller species more because of a disadvantageous surface area to body volume ratio and lower capacity of storing water. Indeed, smaller millipede species usually inhabit colder and less arid places (Enghoff, 1992). In our study, most of the small millipede species recorded live buried in the soil. In this way, they are less influenced by above‐ground management activities, but may be strongly negatively affected by soil degradation and soil contaminants.

Body size is frequently related to the species’ dispersal mode and their ability to disperse (Biedermann, 2002; Jenkins et al., 2007; Kuussaari et al., 2014). In some groups of ground‐dwelling invertebrates, smaller species are mainly passively dispersed among habitats including gardens (e.g., slugs or millipedes transported in soil, attached to garden plants or green waste; Dörge et al., 1999; Stoev et al., 2010). In contrast, larger species of the same groups have to disperse mainly actively through the urban matrix. Thus, larger species may be less likely to reach isolated gardens in the city center. Open gardens with permeable borders enhance colonization by actively dispersing larger species, as shown by the correlation between millipede body size and index of permeable garden border in our study (Figure 3).

Body size did not decrease with increasing urbanization in only two groups: snails and woodlice. In contrast to our observation, Ooms et al. (2020) reported that woodlice body size increased with urbanization in Amsterdam and explained their findings by the advantages of larger body size to reduce water loss under dry and warm conditions (cf. Csonka et al., 2018; Merckx et al., 2018).

## 5 CONCLUSIONS

Our study showed different responses to urbanization by the various groups examined. This confirms that environmental change affects taxonomic groups differently and highlights the need for using multi‐taxa approaches to avoid basing conservation decisions and land‐use managements on the particular response by just a single group. We studied small ground‐dwelling, less conspicuous species, which, however, are of key importance for certain ecosystem functions. Generally, ground‐dwelling invertebrates are little studied and thus not much is known about their population trends and vulnerabilities to environmental change. This precludes management tailored to the needs of such taxonomic groups.

Independent of their location along their rural–urban gradient, domestic gardens are more intensively managed than semi‐natural areas (meadows, forest, and hedges). Therefore, species inhabiting gardens may have traits suitable for the special environmental conditions in this habitat, which in turn may not vary considerably along the rural–urban gradient. The variation in environmental conditions in gardens along the urbanization gradient may thus be lower than those in frequently studied habitats such as urban forests or grasslands. This may also explain why we found few effects of local garden characteristics on the functional diversity of the groups examined. Typical traits of species inhabiting gardens may also be generally helpful in coping with the intense anthropogenic use of green space characteristic of urban areas.

Our findings demonstrate that gardens in highly urbanized areas have similar functional diversity for several organism groups as gardens in rural surroundings. This should motivate urban garden owners to promote native biodiversity in the future.

## CONFLICT OF INTEREST

The authors declare no conflict of interest.

## AUTHOR CONTRIBUTIONS

**Brigitte Braschler:** Conceptualization (equal); Data curation (lead); Formal analysis (lead); Investigation (equal); Methodology (equal); Project administration (supporting); Visualization (equal); Writing‐original draft (lead); Writing‐review & editing (lead). **José D. Gilgado:** Conceptualization (equal); Data curation (supporting); Funding acquisition (supporting); Investigation (equal); Methodology (equal); Project administration (supporting); Visualization (lead); Writing‐original draft (supporting); Writing‐review & editing (supporting). **Hans‐Peter Rusterholz:** Conceptualization (equal); Data curation (supporting); Formal analysis (supporting); Investigation (equal); Methodology (equal); Project administration (supporting); Supervision (equal); Writing‐original draft (supporting); Writing‐review & editing (supporting). **Sascha Buchholz:** Conceptualization (equal); Data curation (supporting); Formal analysis (supporting); Investigation (equal); Methodology (equal); Project administration (supporting); Writing‐original draft (equal); Writing‐review & editing (supporting). **Valerie Zwahlen:** Conceptualization (equal); Data curation (supporting); Investigation (equal); Methodology (equal); Writing‐review & editing (supporting). **Bruno Baur:** Conceptualization (equal); Data curation (supporting); Formal analysis (supporting); Funding acquisition (lead); Investigation (equal); Methodology (equal); Project administration (lead); Resources (lead); Supervision (equal); Validation (lead); Writing‐original draft (lead); Writing‐review & editing (lead).

## Supporting information

Fig S1

Fig S2

Fig S3

Table S1‐S2

## Data Availability

The abundance data per species and garden (presence/absence for plants and ants), the trait values used for each species and the sources used, as well as the landscape variables and garden characteristics for each garden are available from the Dryad Digital Repository (https://doi:10.5061/dryad.fqz612jth).
