# Supplementary figures and images for: Functional diversity and habitat preferences of native grassland plants and ground‐dwelling invertebrates in private gardens along an urbanization gradient
Source: Ecol Evol. 2021 Nov 18;11(23):17043–59. doi: 10.1002/ece3.8343 (PMC8668791; doi:10.1002/ece3.8343)

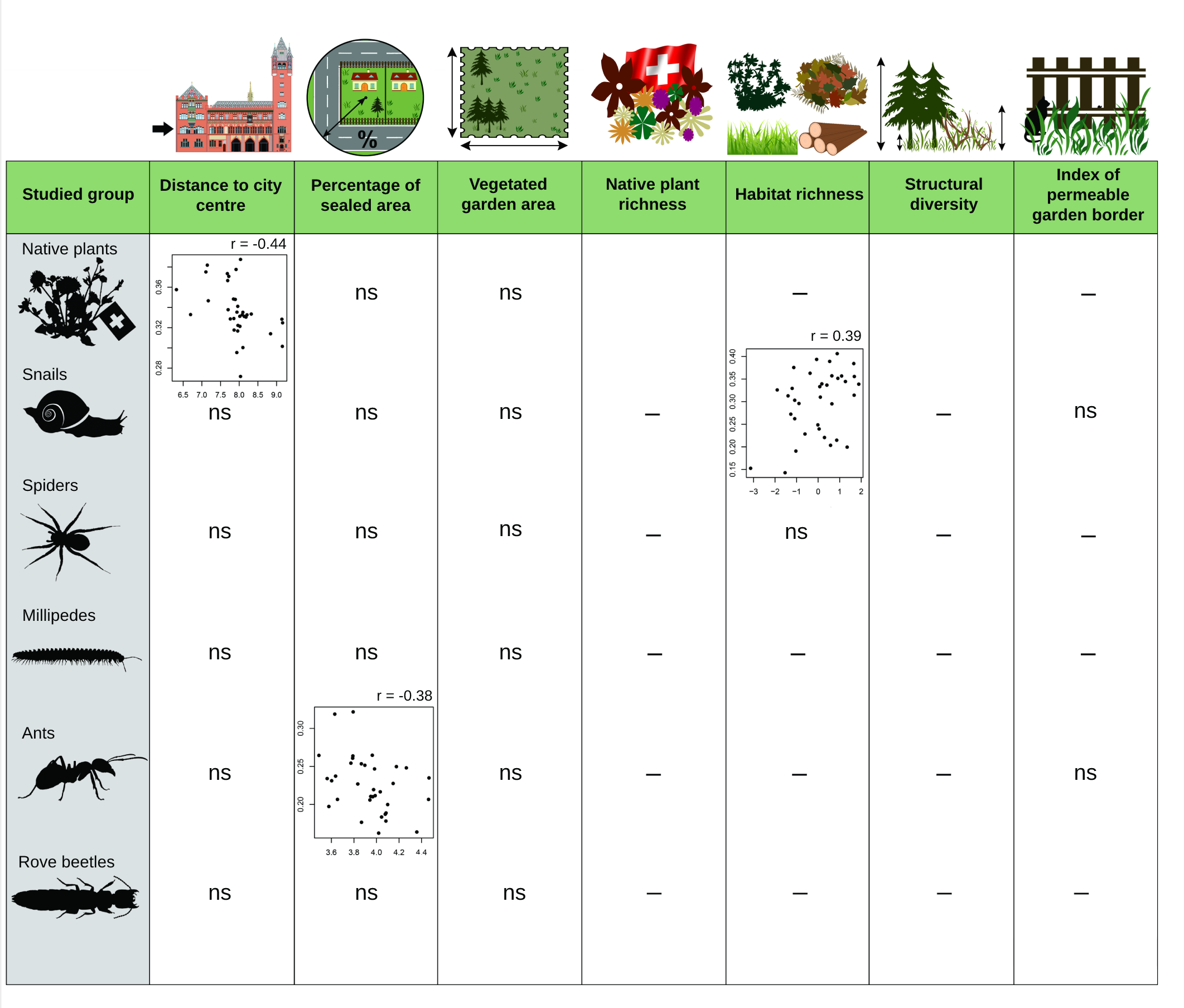

Supplement: Supplementary file 1 — Fig S1 [file ECE3-11-17043-s003.tif]

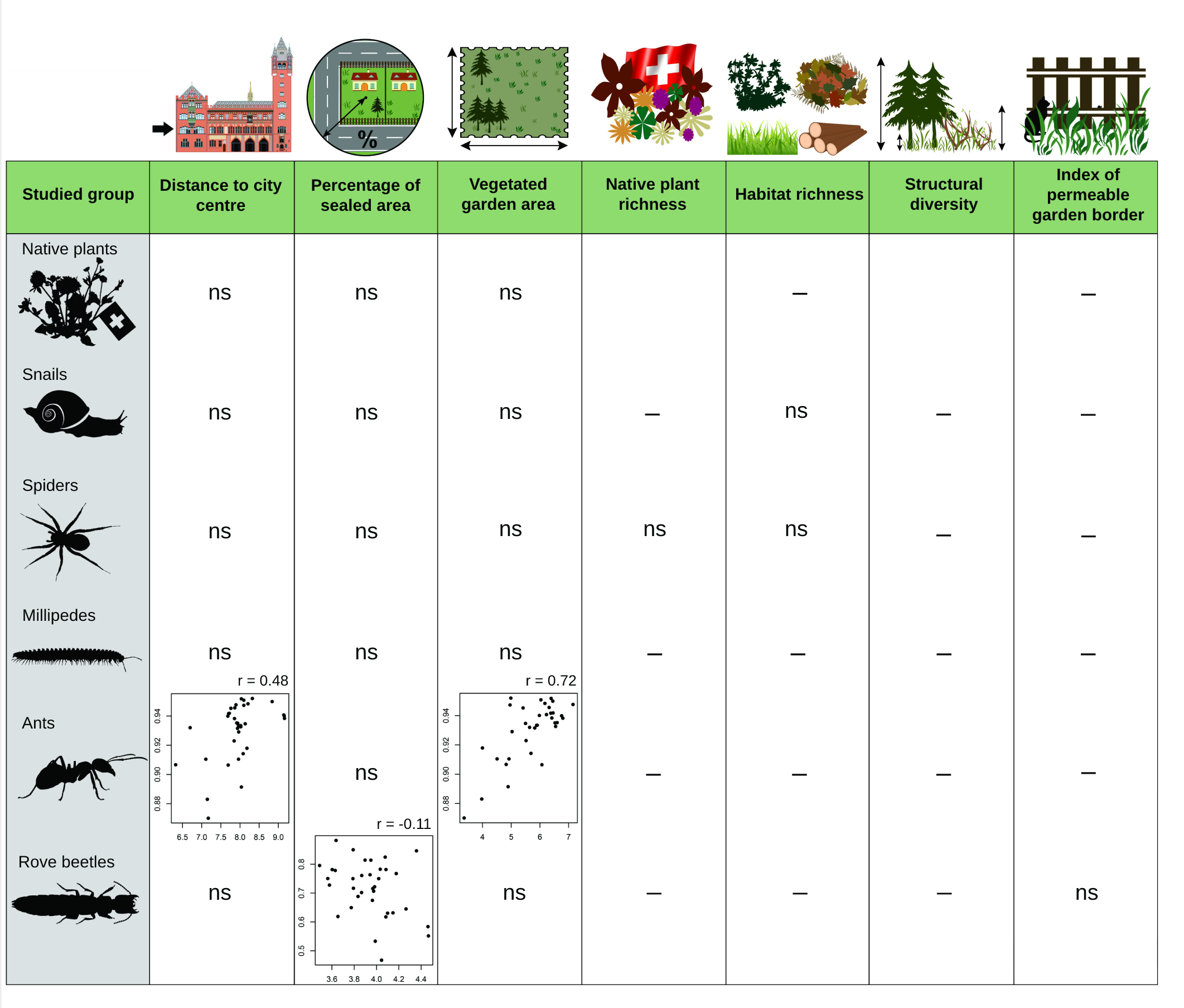

Supplement: Supplementary file 2 — Fig S2 [file ECE3-11-17043-s001.tif]

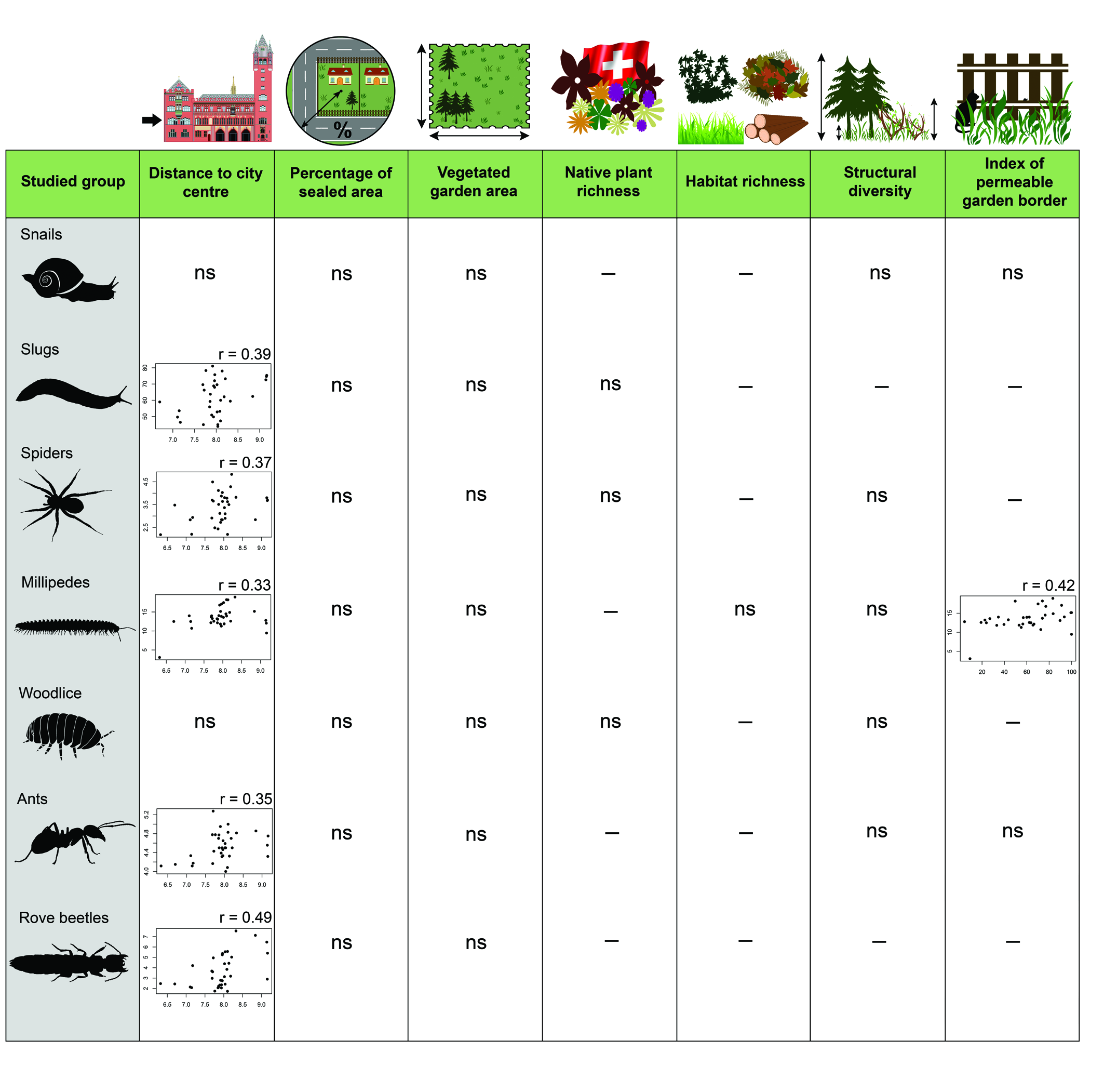

Supplement: Supplementary file 3 — Fig S3 [file ECE3-11-17043-s002.tif]
