# Supplementary material for: Functional diversity and habitat preferences of native grassland plants and ground‐dwelling invertebrates in private gardens along an urbanization gradient
Source: Ecol Evol. 2021 Nov 18;11(23):17043–59. doi: 10.1002/ece3.8343 (PMC8668791; doi:10.1002/ece3.8343)
Supplement: Supplementary file 4 — Table S1‐S2 [file ECE3-11-17043-s004.docx]

**Supplementary Tables**

TABLE S1 Summary of GLM analyses examining the effects of two measures of urbanisation (distance to city centre and percentage of sealed area in the surroundings) and garden size (vegetated garden area) on the proportion of generalist species and of species preferring dry conditions in various organism groups.

|  |  | Proportion generalist species | | | Proportion of species with preference for dry conditions | | | |
| --- | --- | --- | --- | --- | --- | --- | --- | --- |
| Organism group | Variable | df | Chisq | p | df | Chisq | F | p |
| Native grassland plants | Distance to city centre^1^ | 1,33 | 1.93 | 0.16 | 1,33 | 1.29 |  | 0.26 |
|  | Percentage sealed area^1^ | 1,32 | 0.02 | 0.89 | 1,32 | 0.12 |  | 0.72 |
|  | Vegetated garden area^1^ | 1,31 | 0.11 | 0.74 | 1,31 | 1.48 |  | 0.22 |
| Snails | Distance to city centre^1^ | 1,33 | 1.84 | 0.17 | 1,33 | 0.44 |  | 0.51 |
|  | Percentage sealed area^1^ | 1,32 | 0.53 | 0.47 | 1,32 | 0.00 |  | 0.99 |
|  | Vegetated garden area^1^ | 1,31 | 0.25 | 0.62 | 1,31 | 0.03 |  | 0.87 |
| Spiders | Distance to city centre^1^ | 1,33 | 0.31 | 0.58 | 1,33 |  | 0.07 | 0.79 |
|  | Percentage sealed area^1^ | 1,32 | 1.06 | 0.30 | 1,32 |  | 1.42 | 0.24 |
|  | Vegetated garden area^1^ | 1,31 | 2.72 | 0.099 | 1,31 |  | 1.92 | 0.18 |
| Millipedes | Distance to city centre^1^ | 1,33 | 0.93 | 0.33 | nd | nd | nd | nd |
|  | Percentage sealed area^1^ | 1,32 | 0.00 | 0.98 | nd | nd | nd | nd |
|  | Vegetated garden area^1^ | 1,31 | 3.78 | 0.052 | nd | nd | nd | nd |
| Woodlice | Distance to city centre^1^ | 1,33 | 1.65 | 0.20 | 1,33 |  | 0.24 | 0.60 |
|  | Percentage sealed area^1^ | 1,32 | 0.13 | 0.72 | 1,32 |  | 0.02 | 0.88 |
|  | Vegetated garden area^1^ | 1,31 | 0.02 | 0.90 | 1,31 |  | 2.25 | 0.14 |
| Ants | Distance to city centre^1^ | 1,33 | 0.41 | 0.52 | 1,33 | 0.09 |  | 0.77 |
|  | Percentage sealed area^1^ | 1,32 | 1.06 | 0.30 | 1,32 | 0.73 |  | 0.39 |
|  | Vegetated garden area^1^ | 1,31 | 0.08 | 0.78 | 1,31 | 0.32 |  | 0.57 |
| Rove beetles | Distance to city centre^1^ | 1,33 | 2.13 | 0.14 | 1,33 |  | 4.44 | **0.043** |
|  | Percentage sealed area^1^ | 1,32 | 6.45 | **0.011** | 1,32 |  | 0.87 | 0.36 |
|  | Vegetated garden area^1^ | 1,31 | 1.73 | 0.19 | 1,31 |  | 0.25 | 0.62 |

Significant p-values (<0.05) are in bold.

^1^ log-transformed

– variable was excluded from the model by step-wise reduction.

nd no data on preference for dry conditions were available for millipedes.

Quasibinominal was used in spiders, woodlice and rove beetles because of overdispersion. Consequently, a F-value rather than a Chi-square-value is shown.

TABLE S2 Summary of GLM analyses examining the effects of two measures of urbanisation (distance to city centre and percentage of sealed area in the surroundings) and garden size (vegetated garden area), total native plant species richness, habitat type richness, structural diversity of the vegetation, index of permeable border on body size in various organism groups.

|  |  | Body size | | |
| --- | --- | --- | --- | --- |
| Organism group | Variable | df | F | p |
| Snails | Distance to city centre^1^ | 1,33 | 0.01 | 0.90 |
|  | Percentage sealed area^1^ | 1,32 | 0.48 | 0.50 |
|  | Vegetated garden area^1^ | 1,31 | 1.79 | 0.19 |
|  | Total native plant species richness^2^ | – | – | – |
|  | Habitat type richness^2^ | – | – | – |
|  | Structural diversity of the vegetation^2^ | 1,30 | 3.45 | 0.073 |
|  | Index of permeable border | 1,29 | 3.33 | 0.078 |
| Slugs | Distance to city centre^1^ | 1,31 | 5.37 | **0.028** |
|  | Percentage sealed area^1^ | 1,30 | 0.80 | 0.38 |
|  | Vegetated garden area^1^ | 1,29 | 0.01 | 0.91 |
|  | Total native plant species richness^2^ | 1,28 | 1.51 | 0.23 |
|  | Habitat type richness^2^ | – | – | – |
|  | Structural diversity of the vegetation^2^ | – | – | – |
|  | Index of permeable border | – | – | – |
| Spiders | Distance to city centre^1^ | 1,33 | 5.51 | **0.026** |
|  | Percentage sealed area^1^ | 1,32 | 1.10 | 0.30 |
|  | Vegetated garden area^1^ | 1,31 | 0.28 | 0.60 |
|  | Total native plant species richness^2^ | 1,30 | 1.73 | 0.20 |
|  | Habitat type richness^2^ | – | – | – |
|  | Structural diversity of the vegetation^2^ | 1,29 | 3.37 | 0.077 |
|  | Index of permeable border | – | ­ | – |
| Millipedes | Distance to city centre^1^ | 1,33 | 4.75 | **0.038** |
|  | Percentage sealed area^1^ | 1,32 | 0.05 | 0.83 |
|  | Vegetated garden area^1^ | 1,31 | 0.00 | 0.097 |
|  | Total native plant species richness^2^ | – | – | ­ |
|  | Habitat type richness^2^ | 1,30 | 2.87 | 0.10 |
|  | Structural diversity of the vegetation^2^ | 1,29 | 1.08 | 0.31 |
|  | Index of permeable border | 1,28 | 6.28 | **0.018** |
| Woodlice | Distance to city centre^1^ | 1,33 | 0.60 | 0.44 |
|  | Percentage sealed area^1^ | 1,32 | 0.13 | 0.72 |
|  | Vegetated garden area^1^ | 1,31 | 0.31 | 0.58 |
|  | Total native plant species richness^2^ | 1,30 | 1.30 | 0.26 |
|  | Habitat type richness^2^ | – | – | – |
|  | Structural diversity of the vegetation^2^ | 1,29 | 1.76 | 0.19 |
|  | Index of permeable border | – | – | – |
| Ants | Distance to city centre^1^ | 1,33 | 5.34 | **0.028** |
|  | Percentage sealed area^1^ | 1,32 | 0.22 | 0.65 |
|  | Vegetated garden area^1^ | 1,31 | 2.95 | 0.097 |
|  | Total native plant species richness^2^ | – | – | – |
|  | Habitat type richness^2^ | – | – | – |
|  | Structural diversity of the vegetation^2^ | 1,30 | 3.18 | 0.085 |
|  | Index of permeable border | 1,29 | 2.00 | 0.17 |
| Rove beetles | Distance to city centre^1^ | 1,33 | 10.30 | **0.003** |
|  | Percentage sealed area^1^ | 1,32 | 0.40 | 0.53 |
|  | Vegetated garden area^1^ | 1,31 | 0.99 | 0.33 |
|  | Total native plant species richness^2^ | – | – | – |
|  | Habitat type richness^2^ | – | – | – |
|  | Structural diversity of the vegetation^2^ | – | – | – |
|  | Index of permeable border | – | – | – |

Significant p-values (<0.05) are in bold.

Community weighted means were used for body size, except for ants, for which presence/ absence data was used.

^1^ log-transformed

^2^ Due to correlation with total garden size, residuals of the regression of the variable on total garden size were used for analyses.

– variable was excluded from the model by step-wise reduction.

**Figure legends**

FIGURE S1 Effects of urbanisation, garden size, and local garden characteristics, habitat type richness, structural diversity of the vegetation, and index of permeable border on FDis of native grassland plants and five groups of invertebrates. “–“ indicates that this variable was removed from the model in the stepwise procedure, “ns” refers to variables included in the model but that were not significant. Data were transformed as described in the statistical analyses section and Table 2.

FIGURE S2 Effects of urbanisation, garden size and local garden characteristics, habitat type richness, structural diversity of the vegetation, and index of permeable border on FEve of native grassland plants and five groups of invertebrates. For detailed explanations see caption to Figure S1.

FIGURE S3 Effects of urbanisation, garden size and local garden characteristics, habitat type richness, structural diversity of the vegetation, and index of permeable border on body size of seven groups of invertebrates. For detailed explanations see caption to Figure S1.
